# Vascular Lakes in Uveal Melanoma and Their Association With Outcome

**DOI:** 10.1167/tvst.11.3.32

**Published:** 2022-03-29

**Authors:** Hayley Jones, Helen Kalirai, Azzam Taktak, Ke Chen, Sarah E. Coupland

**Affiliations:** 1 Department of Molecular and Clinical Cancer Medicine, Institute of Molecular, Systems and Integrative Biology, University of Liverpool, Liverpool, UK; 2 Department of Engineering, Institute of Risk and Uncertainty, University of Liverpool, Liverpool, UK; 3 Liverpool Ocular Oncology Research Group, University of Liverpool, Liverpool, UK; 4 Liverpool Clinical Laboratories, Liverpool University Hospitals NHS Foundation Trust, Liverpool, UK; 5 Department of Medical Physics and Clinical Engineering, Liverpool University Hospitals NHS Foundation Trust, Liverpool, UK; 6 Department of Mathematical Sciences, University of Liverpool, Liverpool, UK

**Keywords:** uveal melanoma, molecular genetics, prognostication, BAP1

## Abstract

**Purpose:**

Prognostic predictors in uveal melanoma (UM) consist of clinical, histomorphologic, and genetic features. Vascular lakes (VLs) are immature blood vessels within UM with unknown significance for metastatic risk.

**Methods:**

A clinically well-phenotyped cohort of 136 hematoxylin and eosin–stained slides of UM enucleation specimens were retrospectively analyzed on scanned whole-slide images. These were annotated for VL in QuPath, assessing VL number and area. Using SPSS (V27.0), the Mann–Whitney *U* test and Cox regression were applied to evaluate whether there was any correlation between VL number and area within the tumor (VL-TA) compared with other prognostic parameters and patient survival times.

**Results:**

UMs with monosomy 3 (M3) have significant differences in their VL numbers (*P* = 0.008) and VL-TA ratios (*P* = 0.002) compared with disomy 3-UM. Nuclear BAP1-negative (nBAP1^–^) UMs have significant differences in their VL-TA ratio (*P* = 0.002) compared to nBAP1^+^ UMs. Survival times of patients with UM with epithelioid-celled tumors varied depending on their VL-TA ratio (*P* = 0.057). Similarly, in M3-UM, significant differences in survival (*P* = 0.009) were seen in patients, depending on VL number. Finally, patients with UM with shorter overall survival showed significant differences in their tumor VL-TA ratios (*P* = 0.043) and the number of VLs present (*P* = 0.002) than patients with UM who had longer survival.

**Conclusions:**

Our pilot data suggest that VL-TA is an additional poor prognostic parameter in UM.

**Translational Relevance:**

Digital analysis of UM can be easily performed to assess various prognostic parameters. Our pilot study demonstrates that UM-VL could be combined with other parameters to determine metastatic risk of patients with UM.

## Introduction

Uveal melanoma (UM) is the most common primary intraocular malignancy in adults. About half of all patients with UM will develop metastases, most often to the liver, and this is usually fatal. Survival of patients with metastatic UM has not significantly changed in decades, although there are some promising novel therapies on the horizon.^1^

Currently, clinical and histopathologic features are used to predict UM-related mortality: these include tumor size, ciliary body involvement, extraocular extension, mitotic count, epithelioid cell dominance, and closed connective tissue loops, respectively.^2^^–^^7^ Alongside these, chromosomal aberrations and somatic mutations correlate with poor prognostic outcome in patients with UM; these include the loss of one copy of chromosome 3 (i.e., monosomy 3 [M3]), chromosome 8q gains (i.e., polysomy 8q), and inactivating mutations of BRCA1-associated protein 1 (*BAP1*), among others.^2^^,^^8^^,^^9^ The loss of nuclear BAP1 protein expression (nBAP1^–^) in UM cells on immunohistochemistry is often associated with *BAP1* gene mutation. Further, there is a strong correlation between nBAP1^–^ and M3 as well as being associated with other prognostic indicators such as ciliary body involvement, the presence of connective tissue loops, and epithelioid melanoma cells.^10^ Such prognostic indicators in patients with UM allow for them to be stratified into metastatic risk groups, thereby allowing for strategic liver surveillance.^11^

Vascular lakes (VLs) have been well recognized in morphologic studies of UM^12^^,^^13^ and appear as irregular immature intratumoral blood vessels, which lack any endothelial lining. Similar venous structures that lack endothelial lining have previously been described in UM, known as vasculogenic mimicry (VM), which include tubular channels and networks describing patterned matrixes that appear as loops and arcs of vasculogenic vessels mimicking blood vessels.^14^^–^^17^ VLs differ from these structures as they do not loop or arc and appear as standalone structures consisting of pools of plasma and red blood cells. Very little is currently known about VLs within UM: the purpose of this study was to analyze VLs using scanned whole-slide images (WSIs) and digital pathology, to determine whether VLs have any correlation between known prognostic parameters in UM.

## Materials and Methods

### Patient Samples

The study was approved by the Health Research Authority (REC Ref 15/SC/0611) and conducted in accordance with the Declaration of Helsinki. A total of 159 UM specimens and associated data were obtained from the Ocular Oncology Biobank (REC Ref 21/NW/0139).

### Digital Image Analysis

All tumor specimens had been worked up for routine diagnostic histopathology, including staining for hematoxylin and eosin (H&E). Representative H&E-stained UM sections were scanned at 40× magnification using Aperio CS2 (Leica Biosystems, Newcastle-Upon-Tyne, UK) and saved as high-resolution WSIs. The annotation of the scanned WSIs of UM sections was performed using QuPath Bioimage analysis v 0.2.0-m8 (University of Edinburgh, Scotland, UK).^18^ In some large tumors, several H&E sections were scanned; for comparison purposes, the tumor section with the largest number of VLs was assessed. H&E sections were removed from the cohort if they were hemorrhagic or necrotic, because of the difficulty to identify clear VLs.

**Figure 1.**
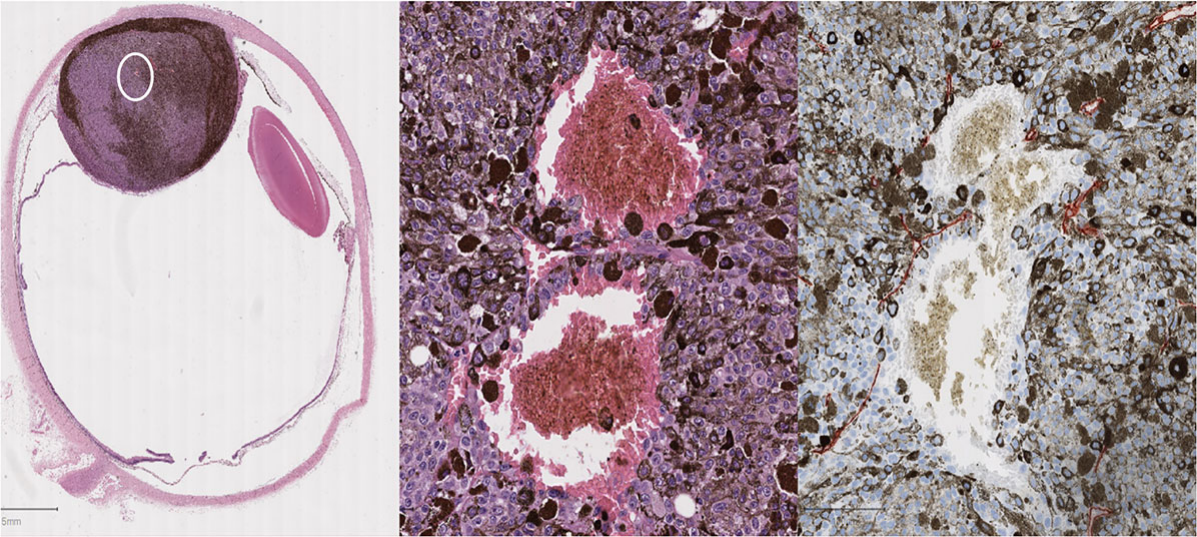
Example of a vascular lake found in uveal melanoma H&E-stained whole-slide image taken from the Liverpool Ocular Oncology Research Group (LOORG) biobank (**A**). Closeup of the vascular lake circled from the H&E slide that is lacking endothelial lining and has some melanocytes diffusing into the lake (**B**). CD34 staining of the same vascular lake from the uveal melanoma slide demonstrating the lack of endothelial lining (**C**).

For reproducibility, a VL was annotated only if it satisfied the following criteria:

• A VL must contain blood and/or plasma.
• Eighty percent or more of the “lake” must lack endothelial lining (Fig. 1).

Where it was uncertain whether a VL had an endothelial lining, immunostaining was undertaken using a CD34 antibody and red chromogen staining (M7165, dilution 1:50, clone: QBEND10; DAKO, Santa Clara, CA, USA).

Annotators were blinded from all patient data, including patient outcome, while the digital analysis was being performed (annotations completed by HJ and SEC).

### Generation of Measurements

From the VLs and tumor annotations, measurements were exported as an ascii text file in a comma delaminated format and imported into Microsoft Excel (Microsoft, Redmond, WA, USA) and combined with the corresponding patient data, producing a new VL-prognostic outcome data set, which consists of a mixture of qualitative and quantitative data. Since the measurements for VL are discrete, the data were converted to continuous; for this, we considered taking the square root of the count of the VL to be a sufficient method of conversion. For the VL–tumor area ratio (VL-TA), the total area of the VL was divided by the total area of the tumor, taken from the annotation measurements.

### Statistical Analysis

Deviation of the prognostic parameters from the normal distribution was statistically significant (*P* < 0.05) after being evaluated by the Shapiro–Wilk test. Therefore, the Mann–Whitney *U* test has been deemed suitable for this study.

From the annotation measurements taken from QuPath of VL numbers, it was determined whether there were statistical differences (*P* < 0.05) for each individual prognostic indicator and the number of VLs present in each tumor and the VL-TA ratio (where prognostic indicator stands for nBAP1 protein loss, M3, polysomy 8q, perforation of Bruch's membrane). After the Mann–Whitney *U* test, the Bonferroni correction method was used to remove the possibility of a false significant test result.^19^

**Figure 2.**
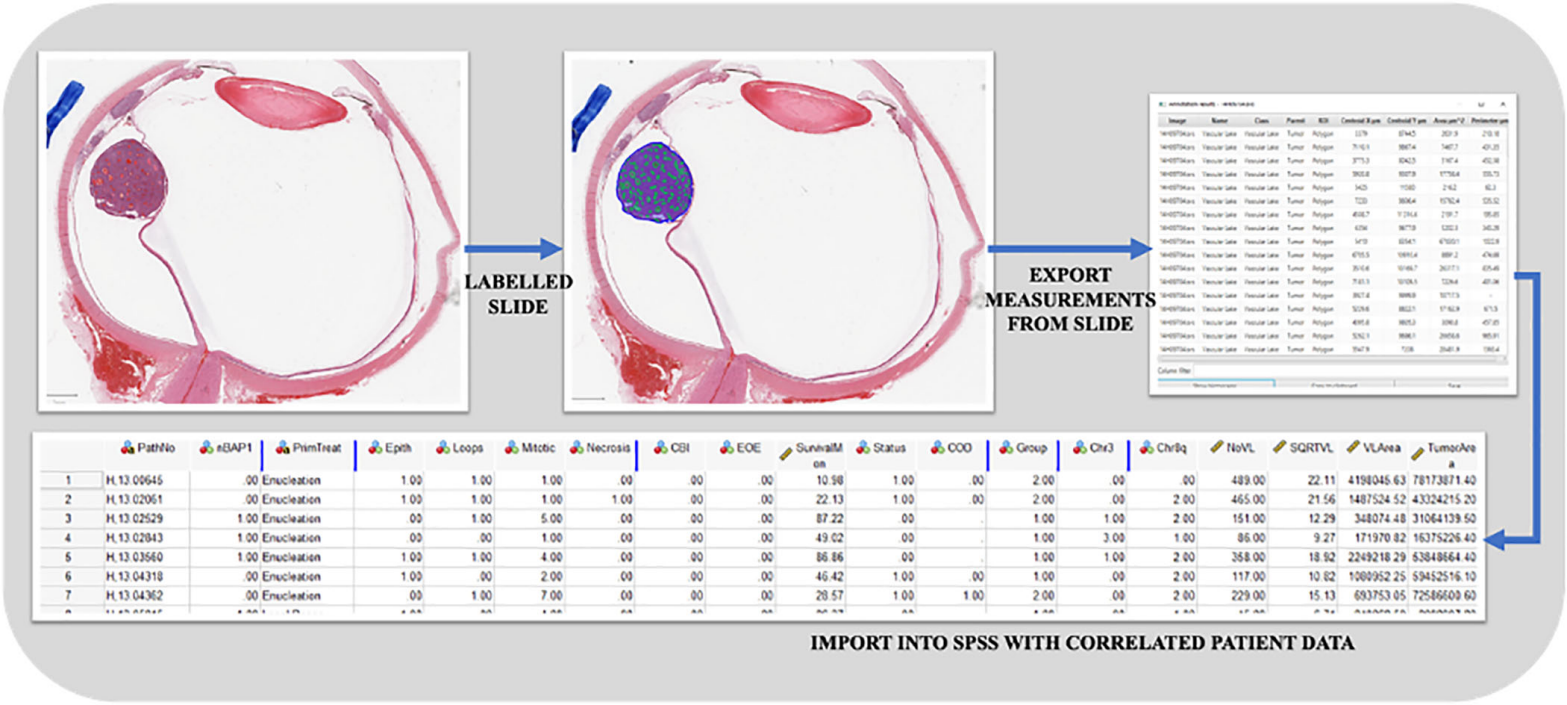
Flow diagram of methods of labeling WSIs and creating combined data in SPSS.

Further analysis was carried out using Kaplan–Meier survival analysis comparing patient survival against the size of the VL-TA using the average VL-TA as a threshold (μ = 0.2). Alongside this, a multivariate model of known prognostic parameters was created to test whether the number and area of VL interaction with known genetic and morphologic indicators affects the survival time of patients. Cox regression with backward selection was chosen, using the likelihood ratio to remove nonsignificant parameters from the model (*P* > 0.1 was the removal criterion). All statistical analysis was computed in SPSS version 27.0 (SPSS, Inc., Chicago, IL, USA), and the workflow can be seen in Figure 2.

## Results

Of the 159 UM cases, 23 samples were removed from the cohort because they were hemorrhagic or too necrotic, leading to the difficulty to identify clear VLs. After this, assessment of a total of 136 WSIs remained for the analysis of VLs against patient outcome and prognostic indicators. The cohort consisted of 57 female patients and 79 male patients, whose ages ranged from 20 to 88 years, with a median age of 64 years. The survival time of the patients ranged from 6 to 387 months, with a median survival time of 61 months. Sixty of the 136 patients are deceased, 40 from metastatic melanoma, 4 from other causes, and 16 whose cause of death is unknown. A full summary of the cohort used for the analysis is in Table 1.

**Table 1.** Patient Cohort Demographic Summary (*N* = 136)

Characteristic	Value
Status at follow-up, *n*	
Alive	73
Deceased	60
Unknown	3
Survival, median (range), mo	61 (6–387)
Gender, *n*	
Female	57
Male	79
Age, median (range), y	64 (20–88)
Morphological, *n*	
Epithelioid cells	89
Closed connective tissue loops	87
Ciliary body involvement	55
Extraocular extension	20
Chromosome status, *n*	
Monosomy 3	87
Polysomy 8q	74
Loss of nuclear BAP1 (nBAP1^–^)	73

The results for each prognostic indicator are summarized in Table 2 (Bonferroni correction: significant if *P* ≤ [0.05/8 = 0.00625]).

**Table 2.** Mann–Whitney *U* Test Results for Prognostic Parameters

Prognostic Parameter	Average No. of Lakes	*P* Value	Average VL-TA Ratio	*P* Value
nBAP1^+^	12.02	0.014	0.03	0.002
nBAP1^–^	9.81		0.01	
Disomy 3	13.06	0.008	0.03	0.002
Monosomy 3	9.74		0.01	
Disomy 8q	10.8	0.834	0.02	0.804
Polysomy 8q	13.17		0.02	
Intact Bruch	9	0.044	0.01	0.024
Perforated Bruch	11.64		0.02	

After applying the Bonferroni correction to the statistical test, our results show that the VL-TA ratio in nBAP1^–^ UM is significantly different from nBAP1^+^ UM (*P* = 0.002). Patients with M3 also showed significant differences in their VL-TA ratio compared with disomy 3 patients (D3) (*P* = 0.002). Patients with nBAP1^–^ UM and M3 had a smaller number of VLs within their tumors; however, these VLs had larger areas than in UM (i.e., smaller VL-TA ratios) with retained nBAP1 expression and D3.

**Figure 3.**
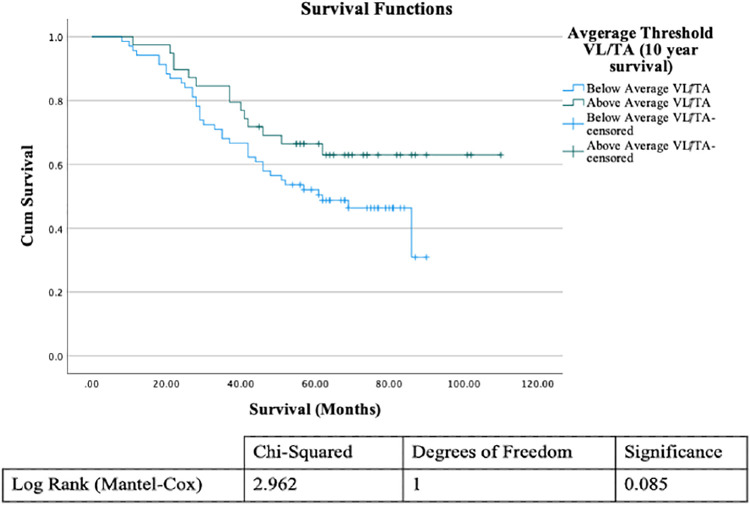
Kaplan–Meier survival curve produced in SPSS of patients’ survival in months using the average area as the threshold for patients’ VL-TA ratio and table of significances from the log-rank survival test.

We also examined whether the survival of patients with UM was affected by the VL-TA ratio within their tumors, using a Kaplan–Meier survival curve (Fig. 3), which shows patients’ survival in months using the average area as the threshold; *P* values were calculated using the log-rank test. The survival curve demonstrates some evidence that patients who had a VL-TA ratio smaller than the mean VL-TA ratio had shorter survival times than patients who had a VL-TA ratio larger than the mean total area ratio, but this result is not significant (*P* = 0.085). Patients with worse prognosis also tended to have larger tumors. The same can be seen with their VLs; patients with worse prognosis tended to have larger VL areas than those with better prognosis. Therefore, for patients with worse prognosis despite their lakes being bigger, their tumors were also much larger, resulting in a smaller VL-TA ratio.

Following on from this, a multivariate analysis was undertaken to see whether the interaction of VL-TA or the number of VLs with prognostic indicators affects patient survival. The data used for the Cox regression are seen in Table 3: this shows how many patient cases were used in the analysis, which cases were removed, and reasons for the removal. The results from the Cox regression can be seen in Table 4. The number of VLs has no affect on surival for patients with D3, but the number of VLs has a significant effect on the survial of patients with M3 (*P* = 0.009). For UM with epithelioid cells, there are survival differences compared to UM without epithelioid cells present (*P* = 0.057) depending on the VL-TA. There are also significant differences for VL number present (*P* = 0.002) and VL-TA ratio (*P* = 0.043) compared to patient survival times.

**Table 3.** Cox Regression Results for Prognostic Parameters

Characteristic	Number	Percent
Cases available in analysis		
Event	53	39.8
Censored	67	50.4
Total	120	90.2
Cases dropped		
Cases with missing values^a^	16	9.8
Total	16	9.8
Total	136	100.0

Dependent variable: survival (months).

a Missing values are from patients without chromosome 8q and and nBAP1 test results, and three patients were lost to follow-up.

**Table 4.** Cox Regression Prognostic Parameter *P* Values

Variable in Cox Regression	
Equation (Final Step)	Significance
nBap1	<0.001
Chr3	0.007
Chr8q	0.10
No VL	0.002
VL/TA	0.043
Epithieliod • VL/TA^a^	0.057
Chr3 • No VL^a^	0.009

a Interaction variable.

## Discussion

The aim of this study was to see if there was any evidence that VLs contributed to worse prognosis in patients with UM. From this novel investigation, we show for the first time in digital pathology that VLs correlate to known histomorphologic and genetic parameters in UM as well as shorter overall survival. To summarize, we defined VLs and assessed their potential use as prognostic indicators in UM: we have clearly shown that VLs are associated with poor prognosis in patients with UM and have also correlated their presence with known prognostic indicators. This study found an association between VL-TA ratio and nBAP1 expression (*P* = 0.002) and chromosome 3 status (*P* = 0.002). M3-UM and BAP1^–^ UM tend to have a smaller number of VLs with larger VL areas (i.e., smaller VL-TA ratios) compared to disomy 3 UM and BAP1^+^ UM, which tend to have a higher number of lakes but with smaller VL areas (i.e., larger VL-TA). A possible reason for this difference could be that these VLs represent immature blood conduits, which even despite increasing tumor growth do not develop a complete endothelial layer. Consequently, as the UM grows, the VLs expand and merge into each other in M3 and nBAP1^–^ tumors, leading to a smaller total number. This may be a reflection of the underlying “immaturity” or stem cell–like nature of the whole tumor. Monosomy 3 or class 2 UM has been demonstrated to comprise less mature stem cell–like tumor cells,^20^^,^^21^ and the same may be applicable to the process of angiogenesis within them. Knowing VLs are correlated to both these prognostic indicators could be particularly useful in laboratories that do not have access to genetic testing, as it allows these laboratories to have some confidence in predicting the UM genetic mutations. When combined with other poor prognostic features, the VLs could be used to identify “high-risk” patients with UM who could have more regular liver surveillance in the attempt to detect the metastases earlier. This is important because this study shows for the first time that VL-TA is correlated with UM patient survival (*P* = 0.043).

Digital pathology allows WSIs to be viewed at high resolutions and provide quicker and easier image analysis, including annotating regions of interest, which are often more accurate and can easily be saved and linked with corresponding patient data more easily than using traditional histopathologic images.^22^^–^^25^ This study is another example of what can be gained from digital pathology, using measurements extracted from WSIs and comparing these with patient data, to find correlations between prognosis and a potential new parameter within UM. Currently, prognosis of patients with UM is based on clinical, anatomic, and morphologic parameters of the tumor cells, combined with their genetic features (e.g., M3, polysomy 8q, the gene expression profile, and the presence/absence of *BAP1* mutations). The latter often leads to loss of nBAP1 expression in UM cells, such that BAP1 immunohistochemistry serves as a surrogate marker for *BAP1* mutations and an indication of chromosome 3 status.

One of the most recent applications of digital pathology in UM and the assessment of a well-established morphologic parameter in these tumors was undertaken by Herrspiegel et al.^26^ These authors examined WSIs of UM and examined the size of tumor cell nuclei and their correlation to patient survival and gene expression.^26^ Their study showed that using digital slides improved the ability to measure the variation in UM nuclei size, which was previously challenging with low reproducibility.^26^

A weakness of this study is the relatively small data set and would benefit from a larger multicenter cohort. Although the Mann–Whitney *U* test can be used on such small data sets, a larger data set would increase the accuracy of the nonparametric test.^27^^,^^28^ If confirmed on a very large cohort, there is the potential of incorporating this parameter into the multiparametric tool, the Liverpool Uveal Melanoma Prognosticator Online (LUMPO3) algorithm, which predicts metastasis in patients with UM with high accuracy.^29^ Furthermore, by perhaps using Z-stacked slices of the UM, the depth of the VLs within the tumor could be determined, to see how this parameter may affect prognosis and to better understand the structure and biology of VLs. Finally, an area of further research is determining the presence or absence of VLs in metastatic UM and observing how they may or may not relate to the degree of maturity of the metastases and the stages of infiltration, as described by Grossniklaus et al.^30^

In conclusion, this study has shown that patients with UM, whose tumors exhibit changes associated with worse prognostic outcome, have significant differences between the number of VLs and/or VL area than in UM without these prognostic indicators. This suggests that larger areas of VLs are correlated to the presence of these poor prognostic indicators and therefore worse patient outcome.
